# Autoencoder-Based Signal Modulation and Demodulation Methods for Sonobuoy Signal Transmission and Reception

**DOI:** 10.3390/s22176510

**Published:** 2022-08-29

**Authors:** Jinuk Park, Jongwon Seok, Jungpyo Hong

**Affiliations:** 1 School of Electrical Engineering, Korea Advanced Institute of Science and Technology, Daejeon 34141, Korea; 2 Department of Information and Communication Engineering, Changwon National University, Changwon 51140, Korea

**Keywords:** sonobuoy, autoencoder, denoising, signal transmission and reception, modulation and demodulation

## Abstract

Sonobuoy is a disposable device that collects underwater acoustic information and is designed to transmit signals collected in a particular area to nearby aircraft or ships and sink to the seabed upon completion of its mission. In a conventional sonobuoy signal transmission and reception system, collected signals are modulated and transmitted using techniques such as frequency division modulation or Gaussian frequency shift keying. They are received and demodulated by an aircraft or a ship. However, this method has the disadvantage of a large amount of information being transmitted and low security due to relatively simple modulation and demodulation methods. Therefore, in this paper, we propose a method that uses an autoencoder to encode a transmission signal into a low-dimensional latent vector to transmit the latent vector to an aircraft or vessel. The method also uses an autoencoder to decode the received latent vector to improve signal security and to reduce the amount of transmission information by approximately a factor of a hundred compared to the conventional method. In addition, a denoising autoencoder, which reduces ambient noises in the reconstructed outputs while maintaining the merit of the proposed autoencoder, is also proposed. To evaluate the performance of the proposed autoencoders, we simulated a bistatic active and a passive sonobuoy environments. As a result of analyzing the sample spectrograms of the reconstructed outputs and mean square errors between original and reconstructed signals, we confirmed that the original signal could be restored from a low-dimensional latent vector by using the proposed autoencoder within approximately 4% errors. Furthermore, we verified that the proposed denoising autoencoder reduces ambient noise successfully by comparing spectrograms and by measuring the overall signal-to-noise ratio and the log-spectral distance of noisy input and reconstructed output signals.

## 1. Introduction

Sonobuoy is a combination of sonar and buoy, and refers to a device that collects underwater information through sound waves. Sonobuoy is a disposable device that is dropped from the maritime patrol to the area of interest. It is designed to transmit the collected underwater signal to the maritime patrol via wireless communication and sink to the sea when the mission is completed. Sonobuoy is divided into passive and active types according to the detection method and the detection range, operating time, and service life vary widely for each product model [1]. Among them, the transmission bit rate of signals is used from hundreds of kbps (kilobits per second) to tens of Mbps (Megabits per second) [2].

**Figure 1:**
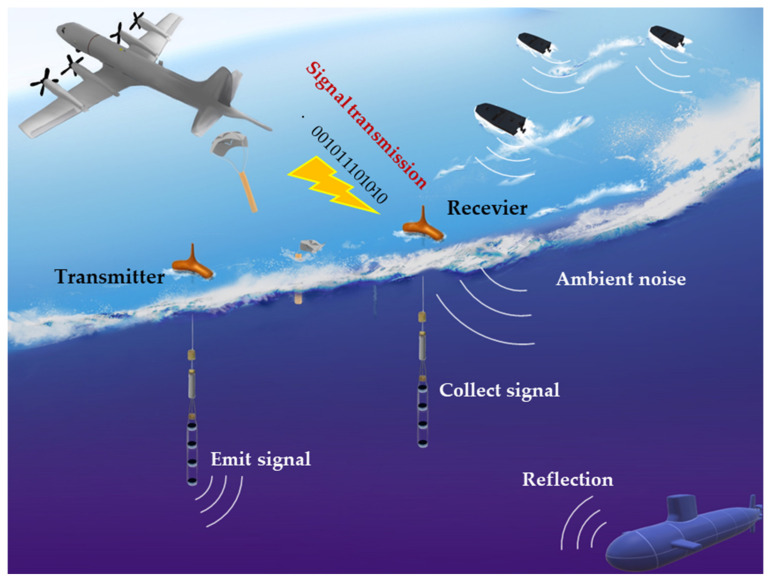
A research conceptual diagram of the bistatic sonobuoy signal transmission and reception.

The sonobuoy may be operated in a monostatic sonobuoy when the active sonobuoy (CASS) or DICASS (Directional CASS) is used alone. It may be operated in a bistatic and multi-static sonobuoy with an explosive sound source or a combination of active and passive sonobuoys [3]. In general, since a bistatic target detection has different positions of a transmitter and a receiver, the detection area is wider and confidentiality is guaranteed compared to monostatic target detection [4]. Figure 1 presents a research conceptual diagram schematically illustrating a bistatic target detection environment using active and passive sonobuoys in anti-submarine warfare (ASW). Since the system of the electronic unit of the sonobuoy cannot perform complex signal processing, sonobuoy inevitably transmits the collected underwater signal to the maritime patrol plane or the ship through wireless communication. The existing signal modulation and demodulation methods used in wireless communication include frequency division multiplexing [5] and frequency shift keying (FSK) [6]. Such a signal modulation and demodulation method have a disadvantage in that a high bit rate is required due to the large amount of information to be transmitted because it is the entire acoustic signal. In addition, since the frequency band of the modulated signal is easily analyzed, the modulation scheme is relatively easy to predict and is highly likely to be demodulated, resulting in low security.

Conversely, using deep neural nets recently, there have been great achievements in various fields, i.e., speech recognition, visual object recognition, object detection, and natural language processing [7]. In particular, autoencoder is an unsupervised learning-based feature extraction technique that can obtain high-level features of input signals by learning and using unlabeled data. It is more practical because it can be applied to a wider range of data than supervised learning, which is expensive to obtain labeled data. The autoencoder mainly consists of encoder and decoder parts, where the encoder yields high-level features, usually called codes or latent variables. These represent input signals compressively well, and the decoder is trained to restore the codes as close as possible to the original inputs. Autoencoder is similar to principal component analysis (PCA) in that it compresses inputs into latent variables by reducing the dimension of data in the encoding process. However, autoencoder is a nonlinear generalization of PCA and presents superior performance to PCA in general [8]. Normally, the structure of the autoencoder is stacked by multiple layers to extract high-level features. Denoising, sparse, and variational autoencoders are developed for further performance improvements in the feature representation [8,9,10,11,12,13,14,15,16,17,18]. According to [10,11], denoising autoencoders learn more key high-dimensional features in the training process by performing denoising tasks on noisy input signals and are known to be superior to the traditional autoencoder [10,11]. Usually, traditional and denoising autoencoders are implemented as under-complete models in which the input dimension is larger than the hidden layer, and as the autoencoder is stacked, the input of the layer is compressed and the number of activations (outputs of each layer) is reduced. Conversely, sparse autoencoders are implemented as over-complete models, which have a larger dimension of hidden layer than the dimension of input, unlike traditional and denoising autoencoders [12,13]. Sparse autoencoders are other methods for extracting interesting structures of data by imposing “sparsity”, which means most nodes are inactive and active nodes exist very rarely, on nodes of layers. However, the disadvantage of sparse autoencoders is the computational complexity of which the activation value must be calculated in advance in order to add sparsity to the cost function [12]. An efficient algorithm to iteratively solve this problem has been proposed [13]. In addition, variational autoencoders are the ones that involve the notion of probability [14,15]. Variational autoencoders are stochastic generative models that model the probability distribution of parameters, whereas the other autoencoders are deterministic discriminative models that model the value itself of parameters [15]. The common and ultimate goals of the above-mentioned methods are to extract high-dimensional features or representations of the input and to improve the performance of tasks (mainly classification) with the features. The results of the autoencoders are used independently or in combination with other methods, i.e., support vector machine (SVM), convolutional neural network (CNN), and Gaussian mixture model (GMM), mainly as a front-end for parameter initialization of supervised learning [16,17,18].

In this paper, a novel approach to apply the under-complete structure of the autoencoder to sonobuoy signal modulation and demodulation for signal transmission and reception in order to decrease the amount of information to be transmitted and increase security. Our contributions are two-fold. First, we propose a method that modulates the transmission signal to a low-dimensional latent vector using an autoencoder to transmit the latent vector to an aircraft or vessel and demodulates the received latent vector to reduce the amount of transmission information and improve the security of the signal. Second, a denoising autoencoder that reduces ambient noises in the reconstructed outputs while maintaining the merit of the proposed autoencoder is also proposed.

## 2. Conventional Sonobuoy Signal Modulation and Demodulation Methods

### 2.1. Frequency Division Multiplexing

**Figure 2:**
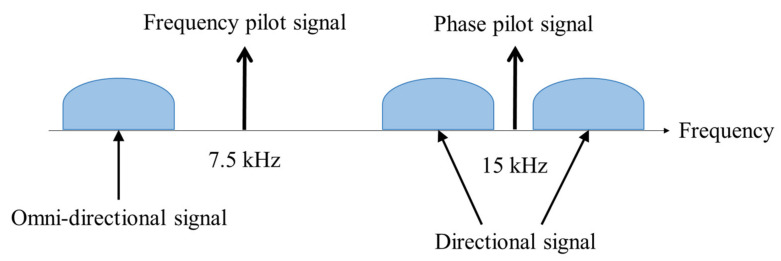
Frequency division multiplexing of DIFAR for signal modulation and demodulation.

Frequency division multiplexing is a method used to transmit multi-channel signals to a single channel, and multi-channel signals are transferred to different frequency bands within a multiplexer. The modulated signals are combined into a single-channel signal through simple addition and then transmitted. Directional frequency analysis and recording (DIFAR) uses frequency division multiplexing and the overall structure is in Figure 2. As shown in Figure 2, DIFAR requires a high bit rate because it transmits the entire signal combined into a single channel, and it is less secure because the transmitted signal is easily distinguishable in the frequency domain.

### 2.2. Frequency Shift Keying

Frequency shift keying transmits information as a frequency change of a carrier signal such as a sine wave. For the simplest binary frequency variation modulation (binary FSK), two signals of different frequencies are used to transmit binary information of 0 and 1. In addition, a Gaussian filter is used for frequency conversion, called Gaussian frequency shift keying (Gaussian FSK), which is a signal transmission method used in various sonobuoy models.

## 3. Proposed Autoencoder-Based Signal Modulation and Demodulation Methods

### 3.1. Autoencoder-Based Signal Modulation and Demodulation Method (General Form)

Autoencoder is a widely used structure in the field of deep learning. It trains the output value of the model to be the same as the input value and forms a symmetrical structure. The structure is largely divided into an encoder, a decoder, and a bottleneck section between the encoder and the decoder. The dimension of the latent vector in the bottleneck section extracted through the encoding is generally much lower than the dimension of the input, and the latent vector reflects the compressed implications of the data. Therefore, the autoencoder mainly serves as a feature extractor using the characteristics of latent vectors to obtain initial weights of other models [16,17,18].

**Figure 3:**
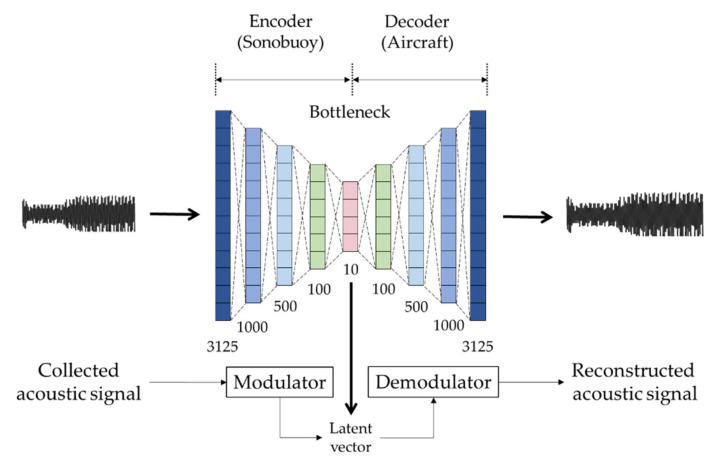
The schematic description of the proposed autoencoder-based signal modulation and demodulation. The trained encoder and decoder are installed on sonobuoy and aircraft, respectively. The main goal of the proposed method is to reconstruct the input signal as close as possible with the latent vector.

In this paper, using the autoencoder structure, latent vectors are extracted by the encoder installed within the sonobuoy and transmitted. The signal processor in the marine patrol reconstructs the original signal from the received latent vectors. The schematic description and the entire training process of the proposed autoencoder-based method are shown in Figure 3 and Algorithm 1, respectively.
**Algorithm 1:** Pseudocode for autoencoder training algorithm1: AE training (e, b, l, E, Δ, θ)2: $x=[x_{1},x_{2},\ldots ,x_{n}]\in R^{N*1}$ is the input signal, in which $x_{i}\in [-1,\;\;1]$
(1≤i≤N) is a single acoustic signal sample3: e is the number of epochs4: l is the learning rate5: b is the batch size6: E is encoder network7: Δ is decoder network8: θ is the network parameters9:    **for** 0 to e **do**10:       $\hat{x}=\Delta (E(x))$11:     $L=\sum_{i=1}^{b}(x-\hat{x}{)}^{2}$12:     L=mean(L)13:    g = gradient of θ14:      **for** θ in θ **do**15:        θ=θ−l∗g16:      **end**17:  **end**

The purpose of this study is to secure the security of the signal using fewer bits than the conventional method when transmitting the sonobuoy signal. The autoencoder judged it to be a suitable model that satisfies both of these purposes. Since the autoencoder trains the same input and output values, it is consistent with the concept of demodulating the transmission signal again. Furthermore, since the latent vectors in the bottleneck section have information that can be restored to the original signal, they can be demodulated to the original signal if only the latent vector and trained demodulator are present, even if the entire signal is not transmitted. By using this, it is possible to transmit signals even in adverse communication environments with a very low bit rate compared to the conventional sonobuoy signal transmission technique. In addition, since all of these transmission and reception processes can only be demodulated by having a basically trained autoencoder model, a third party cannot demodulate into an original signal even if it acquires a transmitting latent vector.

### 3.2. Denoising Autoencoder-Based Signal Modulation and Demodulation Method (Optional Form)

As can be seen in Figure 1, ambient noise exists in the underwater environment [19]. Ambient noise acts as one of the major causes of performance degradation in underwater target detection and identification using signals acquired by sonar and sonobuoy [20]. Therefore, noise reduction algorithms are usually applied as preprocessing to prevent unnecessary performance degradation [20]. In this section, we propose a denoising autoencoder method that can perform the additional ambient noise reduction while maintaining the advantages of the general autoencoder-based method proposed in the above section.

**Figure 4:**
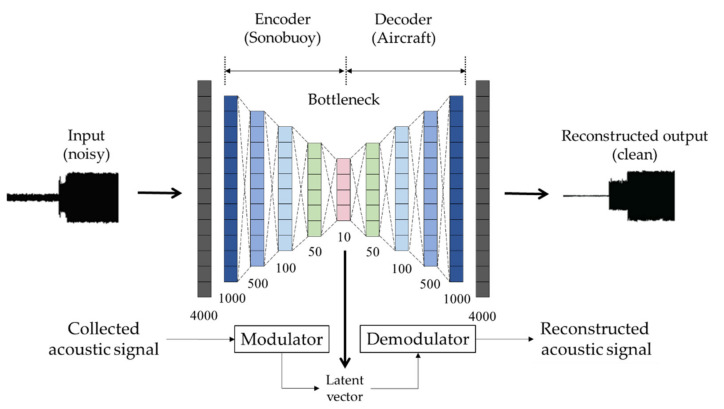
This is the schematic description of the proposed denoising autoencoder-based signal modulation and demodulation. This is an optional form of the general form in Figure 3 for ambient noise reduction. The trained encoder and decoder are installed on sonobuoy and on aircraft, respectively. The main goals of the proposed method are to reconstruct the input signal as close as possible with the latent vector and to reduce ambient noise.

The overall structure of the proposed denoising autoencoder is shown in Figure 4. The denoising autoencoder, like the autoencoder in Figure 3, consists of an encoder, a bottleneck section, and a decoder. However, the output is different in that it yields a noise-removed signal, and the training method for this is different. The entire training process is described in detail in Algorithm 2. The denoising autoencoder is trained with noise-corrupted input data at various signal-to-noise ratios (SNRs) in order to restore the signal of interest even if the input data is distorted or contains a noise. Therefore, using the denoising autoencoder, ambient noise reduction is possible in the transmission and reception process without a separate noise reduction method.
**Algorithm 2:** Pseudocode for denoising autoencoder training algorithm1: DAE training (e, b, l, E, Δ, θ)2: $x=[x_{1},x_{2},\ldots ,x_{n}]\in R^{N*1}$ is the clean input and $z=[z_{1},z_{2},\ldots ,z_{n}]\in R^{N*1}$ is the noise input in which $x_{i}\in [-1,\;\;1]$ and $z_{i}\in [-1,\;\;1]$ (1≤i≤N) are a single acoustic signal sample3: e is the number of epochs4: l is the learning rate5: b is the number of batches6: E is encoder network7: Δ is decoder network8: θ is the network parameters9:   **for** 0 to e **do**10:      **for** j=1 to b **do**11:        r is SNR between $x_{j}$ and $z_{j}$ in dB scale ∈[0,  5,  10,  15]12:        $y_{j}=x_{j}+rz_{j}$13:         ${\hat{y}}_{j}=\Delta (E(y_{j}))$14:         $L=\sum_{j=1}^{b}(x_{j}-{\hat{y}}_{j}{)}^{2}$15:        L=mean(L)16:       g = gradients of θ17:        **for** θ, g in θ, g **do**18:          θ=θ−l∗g19:      **end**20:    **end**21:  **end**

As can be seen in Figure 4, since the network structure is in the under-complete form, the size of the layer is decreasing. As such, it can be transmitted using only a few bits compared to the conventional signal transmission and reception technique; the security of the signal can be guaranteed. In addition, it is possible to obtain an ambient noise-reduced output.

## 4. Experiments with Simulated Data

In this paper, the performance of the autoencoder was verified in a bistatic active sonobuoy environment and the performance of the denoising autoencoder was verified in a passive sonobuoy environment. However, both autoencoder and denoising autoencoder are basically applicable to both active and passive sonobuoy environments.

### 4.1. Experiments for Evaluation of Autoencoder in a Bistatic Active Sonobuoy Environment

#### 4.1.1. Experimental Setup

In this paper, we generate bistatic simulation data in an underwater environment to verify the proposed method. The transmitting signals are generated in two forms: continuous wave (CW) and linear frequency modulation (LFM). The positions of the transmitter and receiver were fixed, and the maximum distance between a target and sonobuoys was limited to 9 km. The target maneuver range was set between 50 m and 150 m. Other detailed conditions for simulation data generation are summarized in Table 1.

**Figure 5:**
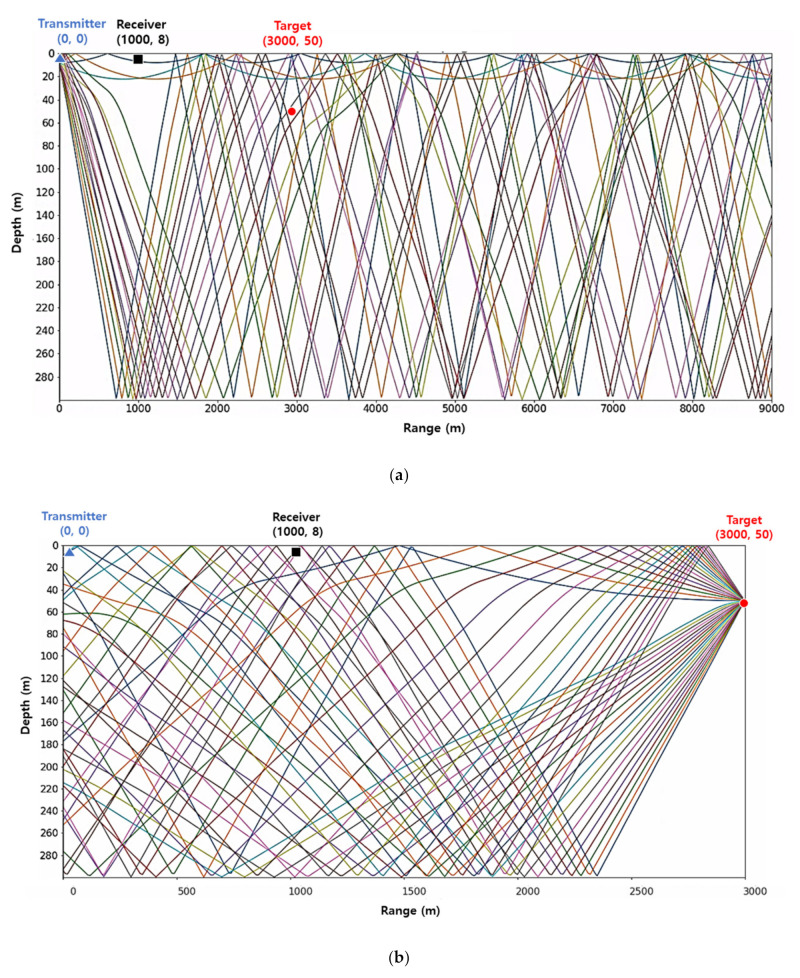
Sound propagation paths samples [range (m), depth (m)] (triangle: transmitter [0,8], square: receiver [1000,8], circle: target [3000,50]): (**a**) Sound propagation paths sample (from the transmitter to the target); (**b**) Sound propagation paths samples (from the target to the receiver).

**Figure 6:**
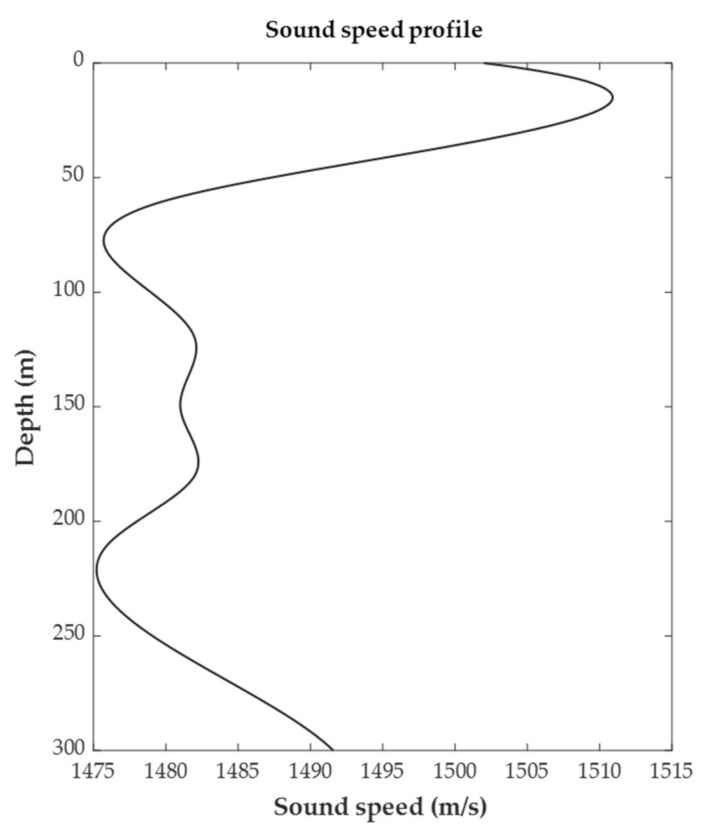
Sound speed profile of the simulated bistatic sonobuoy environment.

Data were generated in a scenario of receiving a pulse signal reflected from a target. Scenarios in which the target location is randomly set are stored as files that are about 10 s long. The total training data were about 50 h, and the evaluation set for evaluating whether the training was converged was about 3 h of data separately from the training data. All data were generated by applying ray tracing [21] as in Figure 5, and the sound velocity profile used for the ray tracing is shown in Figure 6.

Since the sonobuoy signal cannot be modeled with a simple single-layer autoencoder structure, this experiment used a stacked autoencoder to train. Table 2 shows the parameter setting of the model used in the experiment. The parameters were empirically set, and due to the nature of the research data, the result cannot be confirmed only by the loss value, so it should be determined through the restored sample. Although the width and depth of the training layer are not optimal variables, we have failed to model the distribution of complicated input signals when the depth of the layer is too low.

Unlike general audio signals, underwater acoustic signals are very sparse in the frequency domain, thus, we used the time domain acoustic signal as an input for training, which means end-to-end training. Input signals were normalized in the range of −1 to 1 per file, and if the input size of the model was too small, it could not reflect the pulse of one cycle properly, resulting in discontinuity, so it was put into the model in 0.1 s (3125 samples). Between linear layers, ReLU was used as an activation function to reflect the nonlinearity of the input signal, and in the last layer of the decoder, that was used as an activation function to restore the signal to the range from –1 to 1. As a loss function, a mean square error (MSE) between the input and the output signals was calculated for each sample, and the adam optimizer [22] with a learning rate of 0.001 was used.

#### 4.1.2. Experimental Results

To evaluate the original signal restoration performance of the autoencoder, we measured the MSE of the original and the restored spectrograms. The signal used for the evaluation generated 60 s of data not used for model training, and the average energy in the frequency domain was 0.0074. The performances of three autoencoder models (autoencoder I, autoencoder II, and autoencoder III) were measured and summarized in Table 3. The experimental results presented that MSE, which represents a difference from the original signal, had 4.08%, 3.88%, and 3.22%, respectively, compared to the energy average of the original signal. The performance of the model consisting of eight linear layers was the best.

**Figure 7:**
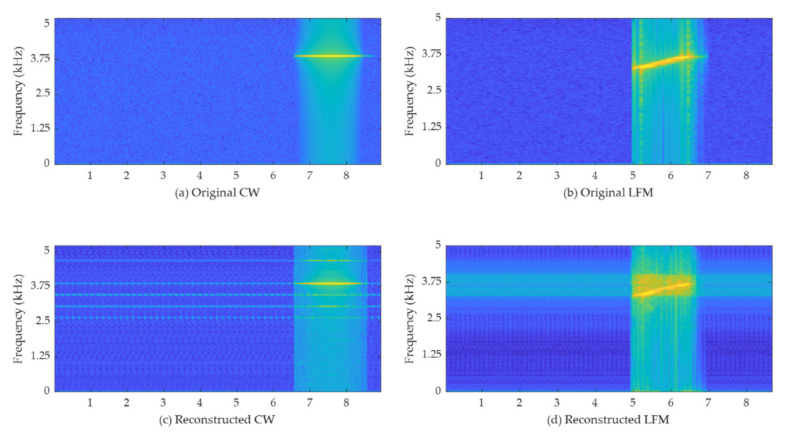
Spectrogram comparison between original and reconstructed signals: (**a**) Original CW; (**b**) Original LFM; (**c**) CW reconstructed by the proposed autoencoder; (**d**) LFM reconstructed by the proposed autoencoder.

Comparing the spectrograms depicted in Figure 7, it can be confirmed that the original signal can be restored by a low-dimensional latent vector with a small artifact. In addition to the frequency band where the echo signal exists, it can be seen that signals such as harmonics are seen or signals such as noise are also present in the signal-absent interval. In the case of the signal-absent interval, it is considered that the magnitude of the data in the interval of the original signal is too small. This results in a noise-like signal in the band in which the echo signal exists due to the bias value of the autoencoder model. In addition, since the proposed method trains in the form of end-to-end without a separate feature extraction process, small value differences in the time domain may appear as noise in the high-frequency band even if the value of the loss function decreases. Nevertheless, both types of signals have been restored very similarly in the frequency bands where echo signals exist. Artifacts generated in the signal absent intervals and bands are negligible compared to the energy of the target signal of interest.

**Figure 8:**
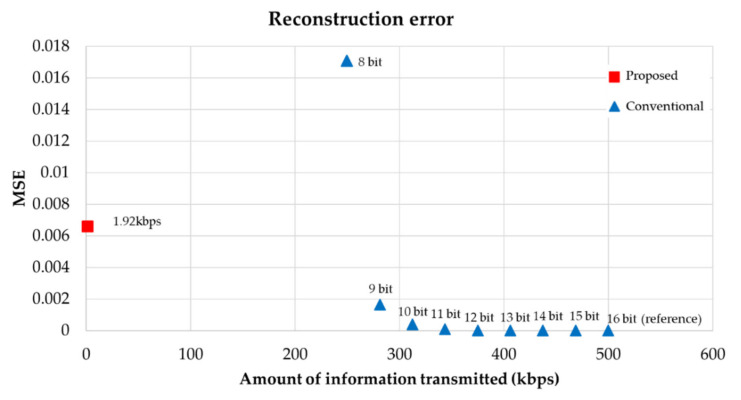
Reconstruction error (MSE) according to the amount of information transmitted. A signal quantized to 16 bits was regarded as a reference signal without errors.

In order to demodulate the original signal, in addition to the latent vector, two values used for normalization must be transmitted. Therefore, if the 10-dimensional latent vector and the size value used for normalization are quantized to 16 bits, the amount of information in the proposed method is about 1.92 kbps. To compare the reconstruction performance, we measured MSE according to the number of quantized bits per sample used in the conventional method [1]. Figure 8 shows the reconstruction error of the conventional and the proposed methods depending on the amount of information required for encoding and decoding. Here, Autoencoder III in Table 3 is used for comparison.

Figure 8 presents that the MSE value of the proposed method (red square) is located between the MSEs of the conventional method (blue triangle) using 8 bits and 9 bits per sample. Considering that the conventional method quantizes to 14 or 16 bits [1], the proposed method relatively presents a large MSE. Therefore, further study to reduce the MSE of the proposed method is necessary. Nevertheless, the reason why we insist that the proposed method is superior to the conventional method is the noticeable reduction in the amount of information transmitted. Consider the cases of quantizing a sampling frequency of 31,250 Hz to 16 bits and 8 bits per sample. These cases require 500 kbps (31,250 samples × 16 bits) and 250 kbps (31,250 samples × 8 bits), respectively. In this paper, we generated a 10-dimensional latent vector every 0.1 s, and store the two-dimensional information that we used for normalization in the training process. Therefore, the proposed method only requires transmitting vectors of 120 dimensions for transmitting a signal of 1 s, not the total number of samples (e.g., 31,250 samples for 31,250 Hz). Compared to the conventional method using 16-bit quantization, the amount of transmission information of the proposed method is 260 times smaller than that of the conventional method. Furthermore, assuming that the amount of transformation information of the conventional method presenting similar MSE performance in Figure 8 is approximately 250 kbps, the amount of transmission information of the proposed method is 130 times smaller than that of the conventional method. This means that the proposed method can encode and decode sonobuoy signals with 130 times less information, which is due to the nature of the latent vector, generated from autoencoder, being represented as a compressed, very high-dimensional feature vector of the input signal. Additionally, owing to the inherent characteristics of the autoencoder, the latent vector can obtain high security that cannot be decrypted without the decoder of the corresponding autoencoder.

### 4.2. Experiments for Evaluation of Denoising Autoencoder in a Passive Sonobuoy Environment

#### 4.2.1. Experimental Setup

In order to verify the performance of the denoising autoencoder, a DIFAR passive sonobuoy detection environment was simulated using the MATLAB Phased-Array System Toolbox [23,24], and detailed experimental conditions are shown in Table 4.

It is assumed that a tonal signal is continuously generated at a target located randomly in the detection range. The signal generated at the target reaches the receiver in consideration of the Doppler effect, the reflection loss of sound waves, and the spreading loss. One file was about 60 s long, a total of 50 h of data were used for training, and 3 h of data were used for evaluation and test.

Like the autoencoder in Table 3, the denoising autoencoder model consists of an encoder and a decoder with multiple layers, and the detailed model structure is shown in Table 5. The input signal was normalized in the range from −1 to 1, chopped by 0.1 s, and inserted into the model for network training. The difference from Table 3 is slight in the number of input samples caused by the different sampling rate, and there is one more layer.

As described in Algorithm 2, the input of the encoder synthesized the clean signal and the white noise with an SNR from 0 to 15 dB. The output of the decoder was trained to reduce the MSE with the clean signal. Through this training method, the noisy signal may be restored as a clean signal for various SNR environments.

#### 4.2.2. Experimental Results

To evaluate the performance of the denoising autoencoder, the spectrogram was subjectively analyzed, and the overall SNR and log spectral distance (LSD) of inputs and outputs were objectively measured [25]. We summarized the noise reduction performance of the proposed denoising autoencoder and the Wiener filter-based sonobuoy noise reduction method [26] in Table 6.

**Figure 9:**
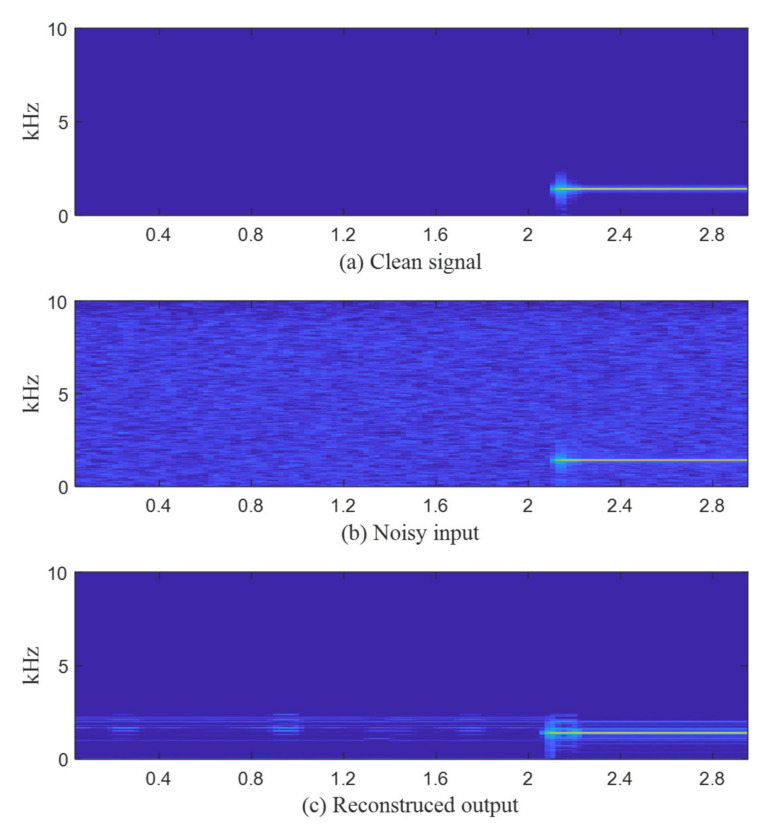
Spectrogram comparison: (**a**) Clean signal; (**b**) Noisy autoencoder input at 5 dB SNR; (**c**) Reconstructed autoencoder output.

In the spectrogram of Figure 9, most of the noises present in the noisy input have been removed from the restored signals. The noise around the signal band remains; however, the level of the remaining noise is negligible.

**Figure 10:**
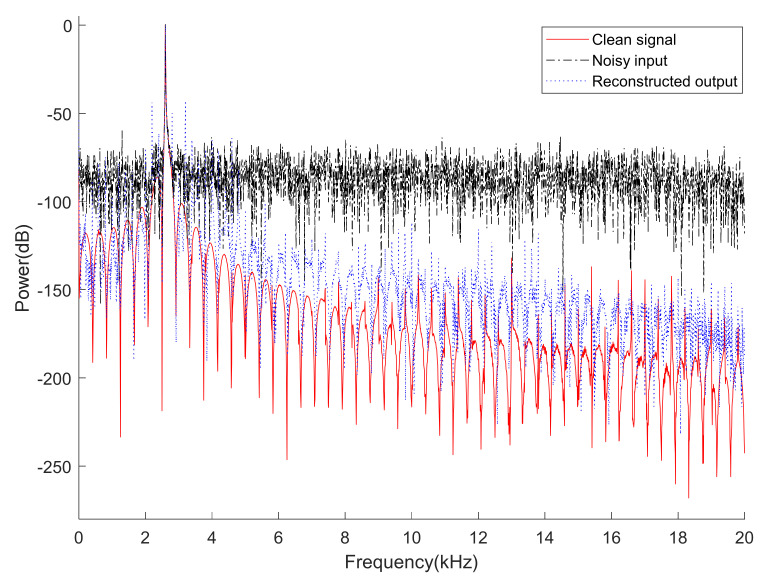
An overlayed frequency analysis of time segments of clean, noisy, and reconstructed signals from 2.4 s to 2.6 s in Figure 9. The “power (dB)” means the square of magnitude of each spectrum represented in decibels. In addition, normalization was performed using each maximum value of clean, noisy, and reconstructed signals for power spectrum comparison. (Red solid: clean signal, black dash-dot: noisy input, and blue dot: reconstructed output).

In addition, an overlayed frequency analysis of time segments of clean, noisy, and reconstructed signals at the 2.4–2.6 s interval in Figure 9 is inserted in Figure 10. Normalization was performed using each maximum value of clean, noisy, and reconstructed signals for power spectrum comparison. In Figure 10, it can be seen that the spectra of the clean signal and reconstructed output are very close by removing the noises distributed in the entire band.

Furthermore, in order to objectively evaluate the noise reduction performance of the denoising autoencoder, overall SNR and LSD for 50 min of test data were measured and summarized in Table 6. Table 6 presented that the proposed method is superior to the conventional method satisfying higher overall SNR and lower LSD simultaneously for all SNR conditions. Through this, it was confirmed that the transmission/reception technique using the denoising autoencoder successfully performs ambient noise removal in the transmission/reception stage without using a separate noise reduction method.

## 5. Conclusions

In this paper, novel sonobuoy signal transmission and reception methods using autoencoders are proposed. Through evaluation, we confirmed that the original signal could be restored from a low-dimensional latent vector by using the proposed autoencoder with approximately 4% errors. We also proposed that the autoencoder shows similar reconstruction performance only using 130 times less information than the conventional method. Furthermore, we verified that the proposed denoising autoencoder successfully reduces ambient noise by comparing spectrograms and by measuring the overall SNR and the LSD of noisy input and reconstructed output signals. The proposed method demonstrates superior denoising performance satisfying higher overall SNR and lower LSD simultaneously for all SNR conditions than the conventional denoising method. However, studies to improve the reconstruction performance by reducing the MSE and to verify the proposed method with real sonobuoy data are necessary and these remain as future works.

## Figures and Tables

**Table 1:** Simulation environmental setup.

	CW	LFM
Center frequency	3500 Hz, 3600 Hz, 3700 Hz, 3800 Hz	
Bandwidth	-	400 Hz
Pulse duration	0.1 s, 0.5 s, 1 s	
Sampling frequency	31,250 Hz	

**Table 2:** Model structure of the baseline autoencoder model.

Encoder (Dim)	Decoder (Dim)
Noisy input (3125)	Latent vector (10)
Linear (3125–1000)	Linear (10–100)
ReLU	ReLU
Linear (1000–500)	Linear (100–500)
ReLU	ReLU
Linear (500–100)	Linear (500–1000)
ReLU	ReLU
Linear (100–10)	Linear (1000–3125)
ReLU	Tanh
Latent vector (10)	Output (3125)

**Table 3:** Comparison of autoencoder models in MSE. Autoencoder III refers to model in Table 2. M denotes 10^6^.

Model	MSE	The Number of Parameters
Autoencoder I	0.00302	6.27 M
Autoencoder II	0.00287	6.66 M
Autoencoder III	0.00238	7.36 M

**Table 4:** Simulation setup for evaluating the denoising autoencoder.

	Tonal Signal
Depth	30, 60, 120, 300 (m)
Detection range	0~15 km
Center frequency	1000~2400 Hz
Sampling frequency	40,000 Hz

**Table 5:** Model structure of the baseline denoising autoencoder model.

Encoder (Dim)	Decoder (Dim)
Noisy input (4000)	Latent vector (10)
Linear (4000–1000)	Linear (10–50)
ReLU	ReLU
Linear (1000–500)	Linear (50–100)
ReLU	ReLU
Linear (500–100)	Linear (100–500)
ReLU	ReLU
Linear (100–50)	Linear (500–1000)
ReLU	ReLU
Linear (50–10)	Linear (1000–4000)
ReLU	Tanh
Latent vector (10)	Output (4000)

**Table 6:** Overall SNR and LSD results at various SNRs. All measures are in dB.

Input SNR		0 dB	5 dB	10 dB	15 dB
Overall SNR	Noisy input	2.24	4.01	6.56	10.35
	Conventional method [26]	4.35	7.13	10.73	15.14
	Reconstructed output	*10.91*	*12.96*	*12.80*	*15.90*
LSD	Noisy input	7.54	7.16	6.78	5.96
	Conventional method [26]	7.05	6.56	6.04	5.52
	Reconstructed output	*3.64*	*3.61*	*3.68*	*3.22*

## Data Availability

Not applicable.
